# Therapies to Suppress β Cell Autoimmunity in Type 1 Diabetes

**DOI:** 10.3389/fimmu.2018.01891

**Published:** 2018-08-16

**Authors:** Charles J. Kroger, Matthew Clark, Qi Ke, Roland M. Tisch

**Affiliations:** ^1^Department of Microbiology and Immunology, University of North Carolina at Chapel Hill, Chapel Hill, NC, United States; ^2^Lineberger Comprehensive Cancer Center, University of North Carolina at Chapel Hill, Chapel Hill, NC, United States

**Keywords:** autoimmunity, diabetes, immunoregulation, immunotherapy

## Abstract

Type 1 diabetes (T1D) is an autoimmune disease that is generally considered to be T cell-driven. Accordingly, most strategies of immunotherapy for T1D prevention and treatment in the clinic have targeted the T cell compartment. To date, however, immunotherapy has had only limited clinical success. Although certain immunotherapies have promoted a protective effect, efficacy is often short-term and acquired immunity may be impacted. This has led to the consideration of combining different approaches with the goal of achieving a synergistic therapeutic response. In this review, we will discuss the status of various T1D therapeutic strategies tested in the clinic, as well as possible combinatorial approaches to restore β cell tolerance.

## Introduction

Type 1 diabetes (T1D) is an autoimmune disease marked by the dysfunction and/or destruction of the insulin-producing β cells found in the pancreatic islets of Langerhans (1–4). T1D most often arises in children but also develops in adults. To compensate for impaired β cell function, T1D individuals require daily insulin therapy. Despite insulin administration, however, establishing optimal glycemic control long-term is often problematic resulting in a number of complications, including retinopathy, nephropathy, vasculopathy, and neuropathy. This morbidity coupled with an increasing incidence of T1D world-wide, underscores an urgent need for effective immunotherapies for T1D prevention and treatment.

The goal of an immunotherapy is threefold: (1) selectively suppress ongoing autoimmunity, (2) reestablish self-tolerance long-term, and (3) preserve acquired immunity. This review will focus on the different strategies of immunotherapy being tested experimentally and in the clinic to suppress β cell autoimmunity, and prevent clinical onset and/or treat T1D.

## Immunopathology of T1D

The loss of β cell tolerance involves both genetic and ill-defined environmental factors (5–16). A strong genetic association with specific human leukocyte antigen haplotypes, coupled with several variants of genes expressed by β cells, T cells, and other immune effectors underscore the complexity of the autoimmune response of T1D (17–23). β cell autoimmunity is typically viewed as a chronic inflammatory response characterized by the progressive infiltration of the pancreatic islets with various immune effectors (24). In the nonobese diabetic (NOD) mouse, a spontaneous model of T1D, islet infiltration is initiated at an early age by macrophages and dendritic cells (DC), CD4^+^ and CD8^+^ T cells, and B cells. This insulitis initially exhibits benign properties with little effect on β cell viability or function. However, at a late preclinical stage, an ill-defined qualitative change occurs within the islet infiltrate, which promotes efficient β cell destruction leading to the onset of overt diabetes. In NOD mice and other rodent models of T1D, pathogenic β cell-specific CD4^+^ and CD8^+^ effector T cells (Teff) are essential drivers of autoimmunity. Diabetogenic CD4^+^ and CD8^+^ T cells target several β cell autoantigens and related peptide epitopes, including proinsulin/insulin, glutamic acid decarboxylase 65 (GAD65), chromogranin A (ChgA), zinc transporter 8 (ZnT8), glucose-6-phosphatase catalytic subunit-related protein (IGRP), and hybrid insulin peptides (HIPs) among others (25–34). It is thought that early in the disease process only few β cell autoantigens and epitopes are targeted by T cells. However, over time additional epitopes within a given autoantigen as well as new autoantigens are recognized. Furthermore, chronic inflammation negatively affects β cells in part by generating neo-autoantigens, such as HIPs, *via* post-translational modification events (32–39). These neo-autoantigens further diversify the diabetogenic response. Together this epitope spread effectively amplifies the β cell-specific T cell response (28, 40–42).

Analyses of human cadaveric T1D pancreases have also demonstrated islet infiltrates consisting of CD8^+^ T cells and macrophages, and to a lesser extent CD4^+^ T cells, and B cells (29, 31, 43–52). However, T1D pancreases have been reported that lack T cell infiltrates suggesting that the immunopathology of human T1D is heterogeneous (53, 54). The prevalence of T cell-independent subsets of T1D is unclear, and thought to be primarily associated with adult T1D onset. On the other hand, evidence indicates that the rapid and severe T1D that develops in children and adolescents is T cell-mediated (44). For instance, recent reports show that childhood onset is marked by a broader and more aggressive β cell-specific T cell response compared to adult T1D (29, 31, 43–52, 55–57). Multiple β cell autoantigens are recognized by human CD4^+^ and CD8^+^ T cells found in peripheral blood, as well as the islets of T1D subjects; many of which are also targeted in the NOD mouse diabetogenic response (e.g., insulin, GAD65, IGRP, and ZnT8) (4, 25, 28, 57).

Pathogenic β cell-specific CD4^+^ and CD8^+^ Teff in NOD and human T1D typically exhibit a type 1 or T helper 1 (Th1) phenotype marked by IFNγ production (47, 58, 59). IL-17-producing CD4^+^ Th17 cells have also been implicated in mediating β cell destruction (60–62). Differentiation and expansion of pathogenic Teff are in part attributed to aberrant peripheral immunoregulation (63–68). An impaired pool of thymic-derived FoxP3-expressing immunoregulatory T cells (Foxp3^+^Treg) has been linked to T1D (68–70). In general, Foxp3^+^Treg play an essential role in maintaining peripheral self-tolerance through cytokine and contact-dependent mechanisms of suppression (71). Decreased survival of islet-resident Foxp3^+^Treg is thought to be a key factor in promoting the progression from benign to pathogenic insulitis in NOD mice (69). Failure to maintain islet Foxp3^+^Treg numbers in NOD mice is due to insufficient local levels of IL-2, a critical cytokine needed for Foxp3^+^Treg survival, fitness, and function (69, 72–74). FOXP3^+^Treg from T1D subjects have defective IL-2 receptor (R) signaling which limits fitness and function of FOXP3^+^Treg (66, 75). Additionally, production of the proinflammatory cytokine IL-21, which is critical for T1D development, can inhibit IL-2 expression by T cells which negatively impacts Foxp3^+^Treg viability and function (76). Human T1D is also marked by deficiencies in non-FoxP3-expressing adaptive (a) Treg. For example, the frequency of β cell-specific IL-10-secreting Tr1 cells is reduced in T1D versus healthy subjects (77–79). In both NOD and human T1D, Teff exhibit a reduced sensitivity to Treg-mediated suppression, which further permits expansion of the diabetogenic Teff pool (63, 64).

**Figure 1:**
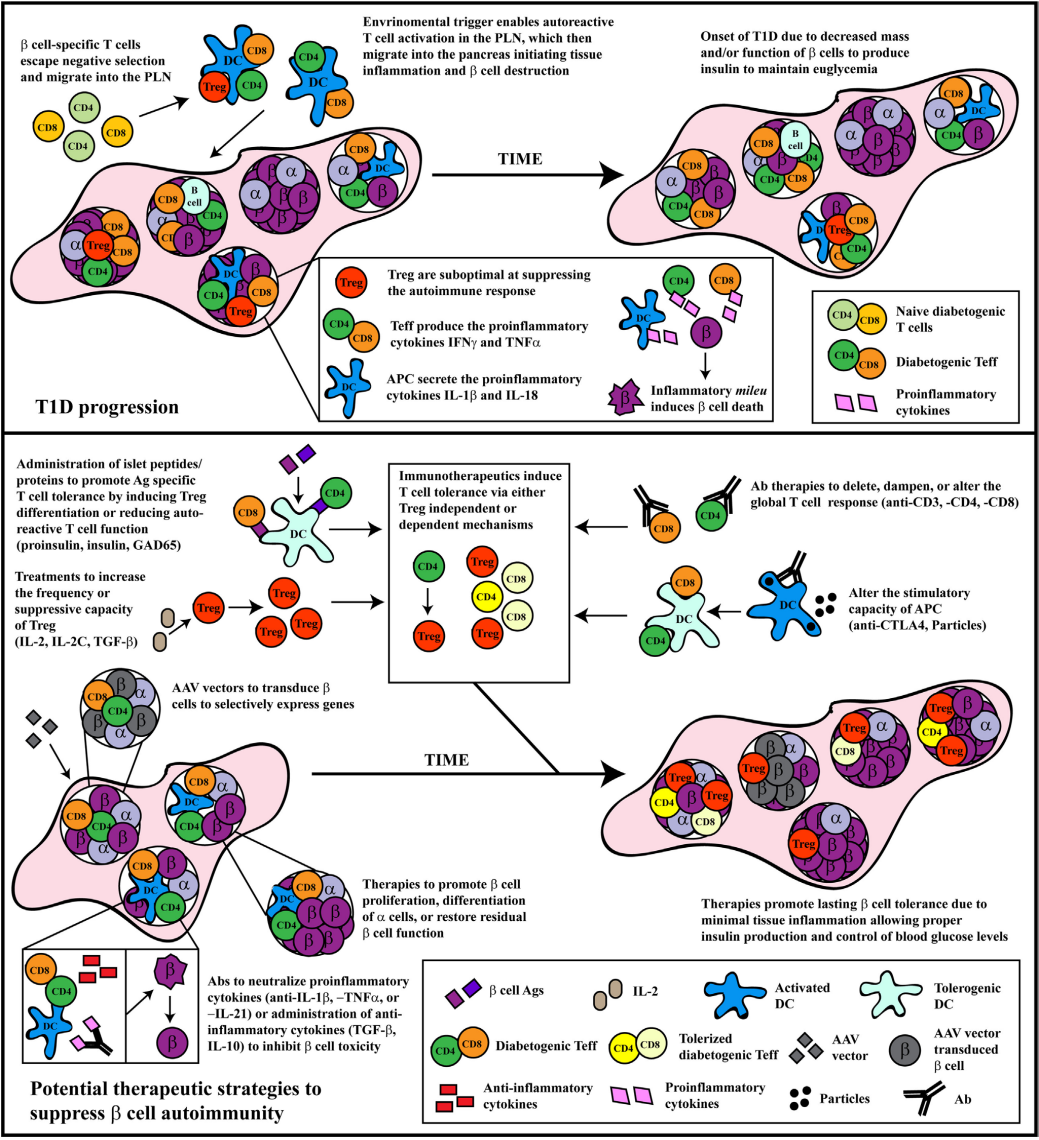
The progression and treatment of β cell autoimmunity. *Top panel*: In general, overt diabetes results from the gradual loss of functional insulin-producing β cells due to the inflammatory environment driven by infiltrating self-reactive T cells and antigen-presenting cells (APC). Although β cell-specific T cell clones are detected in both healthy and type 1 diabetes (T1D) susceptible individuals, a number of factors promote T1D development in the latter population. Decreased efficiency of negative selection in the thymus allows for the increased escape of β cell-specific T cell clones into the periphery. In the periphery, β cell-specific T cells are stimulated in the pancreatic lymph nodes (PLN) by APC derived from the islets, leading to effector T cells (Teff) differentiation due to suboptimal Foxp3^+^Treg suppression. These pathogenic Teff then infiltrate the islets and drive inflammation leading to reduced β cell function and/or survival. *Bottom panel*: Several distinct therapeutic interventions have been tested for the prevention or treatment of T1D. The major approaches have included: administration of β cell autoantigens, expansion of Foxp3^+^Treg, Ab therapies that alter T cell responses, therapeutic reagents that tolerize APC function, neutralization of proinflammatory molecules, or treatments that expand or enhance β cell function and/or survival. Some of these approaches have had clinical, albeit limited, success. Future therapeutic interventions should look toward refinement in the specificity of these treatments, and the development of combinatorial therapies targeting multiple arms of the immune system, as T1D is a multi-pronged autoimmune disease.

Dysregulation among antigen-presenting cells (APC), such as DC, macrophages, and B cells, has also been reported to contribute to T1D (80–85). Although detection of autoantibodies is a key indicator of β cell autoimmunity, B cells are thought to play a critical role in the development of T1D by functioning primarily as an APC (86–88). APC exhibiting proinflammatory properties also skew differentiation of naïve β cell-specific T cells toward pathogenic Teff, as well as amplify islet inflammation and β cell destruction. For instance cytokines, such as IFNγ, TNFα, and IL-1β secreted by islet APC are cytotoxic to β cells *in vitro* (89). The culmination of the adaptive and innate effector immune response within the islets results in β cell destruction/dysfunction and elevated blood glucose levels (Figure 1).

Aberrant peripheral immunoregulation has been the focus of most T1D immunotherapies. Different strategies have attempted to selectively suppress the pathogenic proinflammatory events that affect β cells, while reestablishing protective immunoregulation that persists long-term without altering acquired immunity (Figure 1). Achieving these goals in the clinic, however, has proven to be highly challenging.

## Immunotherapy of T1D

The progression of T1D affords three windows of therapeutic intervention to alter disease outcome (90, 91). The first is during the prodromal stage of T1D, which may persist for a number of years. At-risk individuals with ongoing β cell autoimmunity, identified by serum autoantibodies specific for various β cell autoantigens, are candidates for prevention of clinical onset of T1D (90–92). The second window of intervention is at the time of clinical onset. The goal here is to suppress β cell autoimmunity, rescue residual β cell mass, and ideally reverse clinical diabetes. Early studies provided proof of principle that sufficient β cell mass exists at the time of diagnosis to reverse diabetes (93, 94). Administration of the immunosuppressive drug cyclosporine A (CsA) induces remission in new onset children. However, CsA results in severe kidney toxicity, and once therapy is halted, patients develop recurrent diabetes (95, 96). The third window of intervention is in long-standing T1D patients to protect residual β cell function. Evidence indicates that after T1D onset low but sustained C-peptide levels are observed (93, 94). Therefore, maintenance of a small amount of functional β mass may aid in the control of glycemia as well as limit morbidity.

Two general approaches of immunotherapy have been tested experimentally and in the clinic for the prevention and treatment of T1D. The first approach entails manipulating the autoimmune response independent of β cell specificity. Typically, antibodies (Ab) targeting specific immune effectors or effector molecules have been employed for this approach. The second approach makes use of β cell antigen-specific strategies. Here, β cell autoantigens or corresponding peptides are administered under various conditions. In the following sections, we will discuss different strategies of immunotherapy that are included in these two general approaches, highlighting related strengths and weaknesses (Figure 1).

## Antigen-Independent Immunotherapies

Ab and cytokines have been administered to modulate or block the function of immune effector cells and/or molecules. The general approach is advantageous, since pathogenic effectors can be targeted *en masse*, often resulting in rapid and robust outcomes in experimental settings. However, the limited specificity of these therapeutics can lead to unwanted systemic effects impacting acquired immunity for instance. Nevertheless, a sizable body of work has demonstrated clinical efficacy for certain Ab therapies, whereas promising early results are being seen with cytokine- and cell-based strategies.

### Antibody-Based Therapies

Arguably, the most effective strategy to clinically alter T1D progression has been to target diabetogenic Teff with anti-CD3 monoclonal Ab (mAb) (97, 98). In new onset NOD mice anti-CD3 mAb therapy reverses diabetes in part by driving apoptosis of islet-infiltrating Teff. Engulfment of the apoptotic T cells induces TGF-β secretion by APC that promotes differentiation of Foxp3^+^Treg. Once established this pool of induced (i) Foxp3^+^Treg mediate long-lasting β cell tolerance (99–101). In addition, peripheral Teff exhibit reduced IFNγ production, and increased markers of exhaustion and anergy (102). Interestingly, anti-CD3 mAb therapy given at a preclinical stage fails to prevent diabetes onset in NOD mice (98, 103). This indicates that a given immunotherapy may be effective only at particular stages of T1D progression.

For clinical trials, humanized anti-CD3 mAb hOKT3γ1 (Ala-Ala) (teplizumab) or CCHAglyCD3 (otelixizumab) have been engineered to prevent binding to Fc receptor, and minimize proinflammatory cytokine release by APC (97, 100, 104–112). Anti-CD3 mAb therapy administered to new onset T1D patients reduces the rate of loss of β cell function in the majority of individuals (113). The mechanism of protection is ill-defined, although expansion of CD8^+^ Treg and CD8^+^ Teff exhibiting an exhausted phenotype has been reported (109). Despite this efficacy, diabetes reversal is not achieved, and protection of residual β cell mass is short-term, lasting up to 4 years in some individuals (113). Furthermore, anti-CD3 mAb binding can activate T cells resulting in cytokine release and unwanted inflammation, albeit transient. Moreover, the transient depletion of T cells systemically by anti-CD3 mAb is linked to recurrent viral infections (100, 104). Nevertheless, these findings provide direct evidence that progression of β cell autoimmunity can be modulated by targeting T cells. However, further refinement of the approach is required to enhance β cell protection while minimizing effects on acquired immunity. This may in part be achieved by using anti-CD3 mAb in combination with other therapeutics (114–116). For instance, treatment of NOD mice with anti-CD3 mAb and the β cell trophic growth factor prolactin, increases both diabetes reversal and β cell proliferation relative to anti-CD3 mAb alone (116).

The aim of Ab-based combinatorial strategies is to establish a synergistic or additive effect to enhance both efficacy and safety. An increased tolerogenic response may permit reduced dosing and, therefore, minimize unwanted complications with a given therapeutic. One combinatorial strategy being tested in the clinic is the application of antithymocyte globulin (ATG) and granulocyte colony stimulating factor (G-CSF). Low dose ATG induces apoptosis and transiently depletes T cells, whereas G-CSF promotes mobilization of aTreg and induction of tolerogenic DC. In preclinical studies, ATG alone induces ~30% remission in newly diabetic NOD mice, whereas the combination of ATG and G-CSF increases diabetes reversal >twofold (117). Initial phase I clinical results for low dose ATG plus G-CSF therapy given to recent onset T1D patients indicate that β cell function can be maintained up to 2 years in a group of responders (118). This protective effect correlates with quantitative and qualitative changes in the conventional T cell pool, coupled with an increase in the frequency of FOXP3^+^Treg (118).

Although most T1D immunotherapies have targeted T cells, success has also been demonstrated by altering the B cell response in various autoimmune diseases including T1D, both in mice and humans (88, 119, 120). As previously mentioned, serum autoantibodies indicate B cells also target β cell antigens, including GAD65 and insulin, which serve as a biomarker for individuals with an increased risk for developing T1D (90–92). NOD mice lacking B cells are protected from insulitis and diabetes onset (86, 87). Several molecules, such as CD20, CD22, BAFF, and BCMA have been targeted to modulate the B cell pool (121–125). In different murine models of T1D, anti-CD20 mAb suppresses the progression of β cell autoimmunity as well as reverses diabetes onset, by promoting Foxp3^+^Treg and regulatory B cell populations (121, 122). In recent onset T1D patients, anti-CD20 mAb (Rituximab), which has also been used in the treatment of rheumatoid arthritis (RA), transiently depleted B cells and prolonged β cell function (126, 127). However, this protective effect was only transient, diminishing after 1 year, suggesting brief deletion of B cells alone is not sufficient to restore β cell tolerance.

The depleting effects of a given Ab make dosing problematic, as well as limit the frequency of treatments. On the other hand, nondepleting (ND) Ab provide a strategy to modulate the function of Teff and other immune cells without systemic cell depletion. Human IgG4 is intrinsically ND due to a decreased capacity to fix complement and bind to Fc receptor, and, therefore, is well suited for this purpose. Other IgG isotypes can be engineered accordingly to establish a ND property. A striking example of altering the activity of β cell-specific Teff is observed with the application of ND anti-CD4 and -CD8α Ab. A short-course of ND anti-CD4 and -CD8α Ab induces remission in the majority of new onset diabetic NOD mice without affecting systemic T cell numbers (128–131). Induction of remission is due to Ab-mediated crosslinking of the CD4 and CD8 molecules, which reduces TCR signaling as well as upregulates additional signaling pathways (128, 131–133). The result is suppressed expression of proinflammatory cytokines by Teff combined with the induction of a promigratory phenotype that drives pancreatic egress of Teff (128, 133).

ND Ab and immunoglobulin (Ig) fusion molecules can also serve to block the function of key surface effector molecules. For instance, CTLA-4-Ig (Abatacept)-mediated blockade of the CD80 and CD86 co-stimulatory molecules inhibits APC function and alters the Teff pool in recent onset T1D patients. Abatacept therapy administered monthly for 24 months extends β cell function up to 30 months (134, 135). Examination of peripheral blood shows a decrease in the central memory CD4^+^ T cell population with a concomitant increase in naïve T cells in Abatacept-treated individuals, supporting the notion that Teff expansion is limited (135).

Inhibiting cytotoxic pathways with ND blocking Ab offer another option to protect β cell viability. NOD mice deficient in Fas lack islet-infiltrating effector cells and fail to develop T1D (136, 137). Ab blockade of FasL between 2 and 4 weeks of age prevents T1D in NOD mice. Interestingly, FasL blockade results in the presence of protective IL-10-producing B cells in the pancreas of NOD mice after treatment (138). These results suggest the targeting the Fas–FasL pathway may be a viable therapeutic avenue for the treatment of clinical T1D.

In addition to directly targeting immune effector cells, Ab or Ig-fusion molecules have been used to neutralize secreted effector molecules, such as proinflammatory cytokines. Neutralization of TNFα with several antagonists including mAb (infliximab, adalimumab) and a recombinant TNFα receptor fusion protein, etanercept, have been widely used to treat the autoimmunity driving RA (139). Although TNFα is directly cytotoxic to β cells, its role in the pathogenesis of T1D is controversial. In NOD mice, for instance, TNFα accelerates the progression of β cell autoimmunity when given at a young but not older age (140, 141). In a 24-week clinical study, anti-TNFα therapy in human T1D patients was seen to reduce levels of glycated hemoglobin A1C (HbA1c), a marker indicative of blood glucose values over a 3-month period, as well as increase C-peptide levels suggesting preservation of β cell function (142). In a case report, treatment with infliximab was also found to reduce HbA1c levels, increase insulin secretion, and alleviate insulin resistance in a T1D patient also diagnosed with Crohn’s disease (143). Anti-TNFα therapy, however, fails to prevent the development of T1D in at-risk patients, and may in fact accelerate disease progression (144). These results further underscore how efficacy (or lack of) is dependent on the stage of disease progression at which a given therapeutic is administered.

IL-21 is an appealing cytokine to target and suppress β cell autoimmunity. IL-21 is chiefly produced by T follicular helper cells (Tfh) and Th17 cells, and has diverse roles that include regulating B cell and CD8^+^ Teff function (145–147). The emerging role for both Tfh and Th17 cells in the progression of T1D, and the protective phenotype seen in NOD mice deficient of IL-21 suggests that neutralizing human IL-21 is a promising approach (60, 148–152). Indeed, neutralization of IL-21 with an IL-21R-Ig prevents diabetes in NOD mice (153).

### Cytokine-Based Immunotherapies

Administration of cytokines offers an approach to reestablish peripheral immunoregulation and β cell tolerance. One such strategy currently being investigated is administration of low dose IL-2 (65, 154–156). Conventional CD4^+^ and CD8^+^ T cells but not Foxp3^+^Treg are producers of IL-2, which plays a central role in driving both pro- and anti-inflammatory responses (157, 158). IL-2 for instance is needed for conventional T cell activation and expansion, and IL-2 drives proinflammatory responses by other immune effectors (158). IL-2 also plays an essential role in Foxp3^+^ Treg survival, expansion, and suppressor function (159–161). Constitutive expression of the high affinity IL-2R (CD25) allows Foxp3^+^Treg to out-compete conventional T cells for limiting amounts of IL-2. The latter provides rationale for using low dose IL-2 to preferentially affect Foxp3^+^Treg versus conventional T cells (154). Indeed, low dose IL-2 has been effective in the clinic for the treatment of graft-versus-host disease, and systemic vasculitis (162, 163). In NOD mice, low-dose IL-2 prevents clinical onset and reverses diabetes *via* an expanded pool of Foxp3^+^ Treg in the islets and draining pancreatic lymph nodes (69, 164, 165). Similarly, low-dose IL-2 in combination with rapamycin in recent onset T1D patients increases the frequency of Foxp3^+^Treg in blood (166). However, these patients also exhibit an accelerated rate of β cell loss (166), suggesting an enhanced pathogenic response, and highlighting the key problem of administering a cytokine with pleiotropic effects (167, 168).

Different strategies are being developed to enhance the efficacy of IL-2 (and other cytokines), while avoiding unwanted systemic effects (169, 170). One approach is to promote selective binding of IL-2 to Foxp3^+^Treg *via* IL-2-Ab complexes (IL-2C) (170–172). Targeting particular epitopes on IL-2 with anti-IL-2 Ab can favor binding to the high affinity IL-2R constitutively expressed by Foxp3^+^Treg (173). Administration of IL-2C readily expands Foxp3^+^Treg in mice and prevents autoimmunity (170–172). While promising, polyclonal expansion of Foxp3^+^Treg by IL-2C may compromise protective immunity against pathogens.

An additional strategy to minimize the systemic effects of IL-2 while expanding β cell-specific Foxp3^+^Treg is to target cytokine expression to β cells *in vivo*. Adeno-associated virus (AAV) vectors have been used to deliver and target expression of an insulin promoter-driven IL-2 transgene (AAViP-IL2) to β cells *in vivo* (72–74, 174). In general, AAV vectors are appealing for *in vivo* gene delivery due to limited immunogenicity, lack of integration into the genome, and efficient transduction of non-proliferating cells (175). Notably, treatment of NOD mice at a late preclinical T1D stage with AAViP-IL2 results in the expansion of islet-resident Foxp3^+^Treg, suppression of β cell-specific Teff, and prevention of diabetes onset (72–74). AAV vectors can be further exploited by co-delivering genes encoding other anti-inflammatory cytokines (e.g., IL-10, TGFβ1, and IL-35) and/or pro-survival proteins to enhance both the tolerogenic effect and maintenance of β cell mass *in vivo* (72, 174, 176–179).

### Foxp3^+^Treg-Mediated Therapy

An alternative strategy to manipulate the Foxp3^+^Treg population *in vivo* is to transfer Foxp3^+^Treg that have been expanded *in vitro* (180–182). The approach is effective in preventing diabetes onset in NOD mice (183). However, *in vitro* expansion of a homogeneous and stable pool of Foxp3^+^Treg, particularly when starting from relatively few cells, has been technically difficult. Expansion protocols are being devised using drugs such as rapamycin to prevent outgrowth of Teff, as well as DNA methyltransferase and histone deacetylase inhibitors to enhance Foxp3^+^Treg stability (184, 185). Despite these hurdles, clinical studies have shown Foxp3^+^Treg therapy is well tolerated and therapeutic efficacy is observed for various diseases (180, 181). Furthermore, a phase I trial has demonstrated that *in vitro*-expanded Foxp3^+^Treg persist long-term and exhibit a stable phenotype when transferred back into T1D subjects (180). Notably, loss of C peptide is also limited in some patients. These results provide justification for additional clinical studies to directly assess the efficacy of Foxp3^+^Treg transfer for T1D treatment.

### Microbiome Interventions in T1D

In recent years, the importance of the gut microbiota in maintaining the normal function of the immune system has emerged (186). Reports have documented that changes in gut microbiota can markedly influence T1D development, and that differences in the colonizing microflora contributes to variations in T1D onset among NOD mouse colonies (187, 188). Toll-like receptors (TLRs) play a crucial role in the innate immune system’s recognition of common bacterial and viral components. TLR signaling triggers APC maturation and production of proinflammatory mediators (189). One of the major TLR signaling adaptor molecules driving APC maturation is MyD88, which activates several downstream signaling pathways (190). NOD mice lacking MyD88 expression and housed under normal specific-pathogen-free conditions exhibit a reduced T1D incidence. In contrast, MyD88-deficient NOD mice housed in germ-free conditions develop robust diabetes (16). Interestingly sex differences in the gut microbiota also influence T1D susceptibility. For example, young female NOD mice receiving gut microbiota from male NOD mice exhibit a reduced T1D incidence (191). These results demonstrate that the gut microbiota can have a protective role against autoimmunity, and specifically T1D. Furthermore, differences in the biodiversity of the gut microbiome are seen between T1D patients and healthy individuals (192–196). Similarly, the microbiome within the gut of T1D infants fails to diversify during development in comparison to healthy individuals, potentially due to hyperglycemia (197). Here, changes in the production of microbial metabolites such as short-chain fatty acids found in the colonic lumen and peripheral blood are thought to impact immunoregulatory networks. Indeed, a recent study showed that diets that enhance production of acetate and butyrate by the gut microbiome, effectively prevent diabetes in NOD mice (198). Increased acetate is seen to reduce β cell-specific Teff, whereas elevated butyrate expands Foxp3^+^Treg. Together these results indicate an association between modified gut microbiota and compromised self-tolerance. Manipulating the gut microbiota may prove to be an effective adjuvant therapy to limit the progression of β cell autoimmunity in at-risk individuals.

## Antigen-Dependent Immunotherapies

The goal of antigen-based therapies is to selectively tolerize the autoreactive Teff pool and induce or expand autoantigen-specific Treg (199–202). The approach is appealing, since β cell antigen vaccination is expected to have no effect on acquired immunity. Teff are tolerized *via* whole antigen or peptide by various mechanisms, including: (i) clonal anergy, (ii) clonal deletion, or (iii) exhaustion. This has classically been achieved by administration of high doses of soluble antigen or peptide. On the other hand, antigen vaccination has been used to induce differentiation of naïve β cell-specific T cells into aTreg, including iFoxp3^+^Treg. Depending on the conditions of antigen vaccination, distinct subsets of aTreg can be induced. Expanding the aTreg pool is advantageous, since local cytokine-mediated suppression is independent of Teff antigen-specificity; this bystander-mediated suppression also can downregulate the activity of proinflammatory APC. Success of either tolerizing Teff or inducing/expanding Treg is dictated by multiple factors including dose, the frequency, and route of antigen vaccination. Also important is the context in which the antigen is delivered. Antigen administration in the context of an adjuvant, microparticles, or DC can significantly alter the nature of the T cell response. The most critical factor determining efficacy, however, is the identity of the autoantigen (or peptide) used for vaccination. To effectively block the progression of β cell autoimmunity, prevalent, or immunodominant Teff clonotypes need to be targeted. Ongoing efforts defining the specificity of islet-resident Teff are expected to identify immunodominant Teff clonotypes (31). Furthermore, promising antigen-based immunotherapies are exploiting advancements in targeted drug delivery systems to effectively tolerize Teff and promote Treg-mediated suppression.

### β Cell-Autoantigen Vaccination

Early clinical studies have highlighted the difficulty in establishing a protective, β cell-specific T cell response *via* autoantigen vaccination (203). The Diabetes Prevention Trial-type 1 tested parenteral insulin delivery *via* intravenous (i.v.), subcutaneous (s.c.), and oral routes in at-risk individuals (202, 204–206). No significant difference, however, was detected in the frequency and onset of diabetes in the treatment and control cohorts. Similarly, phase II and III studies of s.c. vaccination of aluminum hydroxide (alum)-formulated GAD65 to new onset T1D patients had no marked effect on C-peptide secretion over time (207, 208). Alum as an adjuvant is included due to a potent capacity to induce IL-4-secreting Th2 cells, which in turn can block the differentiation of pathogenic IFNγ-producing Teff (209, 210).

Additional studies have tested insulin and proinsulin peptides to restore β cell tolerance in human T1D patients. An altered-peptide ligand of the insulin B chain 9–23 epitope administered s.c. to recent onset T1D subjects reduces peptide-specific IFNγ-secreting Teff, while elevating Th2-like aTreg (211, 212). Nevertheless, no clinical benefit was detected, suggesting that altering insulin B chain-specific Teff reactivity alone is insufficient to induce robust β cell tolerance. Clinical trials assessing intradermal vaccination of a natural peptide derived from proinsulin (C19-A3) have also been carried out (213, 214). Vaccination with low (30ug) versus high (300 µg) doses of C19-A3 increases the frequency of peptide-specific CD4^+^ Tr1 cells in long-standing T1D subjects (213). A similar increase in CD4^+^ Tr1 cells is detected in newly diagnosed T1D subjects vaccinated with C19-A3 (214). Notably, FOXP3^+^ Treg are also increased, and evidence suggests that residual β cell function is preserved in some individuals (214). Treatment with a pool of peptides derived from additional β cell autoantigens may promote a more robust tolerogenic effect on Teff, as well as enhance induction and/or expansion of the aTreg pool.

### Strategies to Enhance the Efficacy of β Cell-Autoantigen Vaccination

As noted above the context of autoantigen vaccination is critical for determining the nature and magnitude of the induced T cell response, and in turn therapeutic efficacy. Various strategies are being developed to better “tailor” the antigen-specific T cell response. One such approach has been the application of plasmid DNA (pDNA) vaccination. Injection of soluble pDNA or *via* gene gun delivered pDNA-coated particles results in significant levels of transgene expression lasting several weeks *in vivo* (215, 216). Co-vaccination of pDNA encoding β cell autoantigens and anti-inflammatory cytokines both induces protective β cell-specific aTreg, and prevents diabetes onset in NOD mice (174, 217–220). In a T1D clinical trial, patients received intramuscular injections of pDNA encoding proinsulin over a 12-week period. C-peptide levels were maintained up to 3 months after treatment, which corresponded with a reduced frequency of proinsulin-specific CD8^+^ T cells in peripheral blood (221). Notably, the frequency of CD8^+^ T cells specific for foreign antigen remained unperturbed, illustrating a key strength of β cell-autoantigen vaccination.

An additional approach to enhance the efficacy of β cell-autoantigen vaccines is the use of small molecule particles (222, 223). Typically, injected β cell antigens and other soluble reagents have a short half-life *in vivo*, which can be greatly enhanced by encapsulation into a particle. Particles are generated using different compounds, including poly(lactide-co-glycolide) and acetalated dextran (222, 224). The particle size and composition can be readily manipulated to determine cellular uptake and cargo release *in vivo* (225). Some studies have included cell-specific Ab or ligands to target particles to a desired cell population, and avoid off-target effects (222, 226–228). However, most treatments utilize the natural phagocytic function of DC and macrophages to engulf the injected particles. In this way, a tolerogenic APC phenotype can also be established *in vivo* by encapsulating various immunomodulatory agents such as the aryl hydrocarbon receptor ligand 2-(1′H-indole-3′-carbonyl)-thiazole-4-carboxylic acid methyl ester, rapamycin, dexamethasone, cytokines, and/or antisense oligodeoxynucleotides (222, 223, 229–232). These APC upon presenting the loaded antigen more readily tolerize Teff and/or induce aTreg (229–232).

As opposed to delivering a cargo to APC, nanoparticles have also been used as surrogate APC (233, 234). Here the surface of nanoparticles is complexed with MHC class II (MHCII) molecules tethered to a β cell-derived peptide (234). These MHCII-peptide-coated nanoparticles promote differentiation of CD4^+^ Teff into Tr1 cells that are readily expanded, and in turn drive diabetes remission in NOD mice (234). Another approach has exploited the tolerogenic properties of apoptotic cells. Uptake of apoptotic bodies by immature DC and macrophages blocks subsequent APC maturation and promotes upregulation of TGFβ1 (235). Accordingly, i.v. injection of 1-ethyl-3-(3 dimethylaminopropyl) carbodiimide (ECDI) fixed splenocytes loaded with autoantigen suppresses experimental autoimmune encephalomyelitis, a murine model of multiple sclerosis (MS) (236). Currently, this approach is being tested in clinical trials in which MS patients receive an i.v. transfusion of ECDI-fixed peripheral blood mononuclear cells loaded with MS-relevant peptides recognized by CD4^+^ T cells (237, 238).

## Summary

Type 1 diabetes is a complex disease driven by pathogenic adaptive and innate responses leading to the dysfunction and destruction of β cells (1–4). Although disease immunopathology is heterogeneous, T cells are considered to be the key mediators of β cell destruction/dysfunction for the majority of cases of human T1D (57). Consequently, T cells have been the focus of most strategies of immunotherapy. Suppression of pathogenic β cell-specific T cell reactivity in the islets long-term, while preserving acquired immunity is the ultimate goal for a given immunotherapy (Figure 1). Achieving this goal in the clinic, however, has been elusive. To date, the most effective strategies have been broadly acting on the majority if not all T cells although protection has also been demonstrated by targeting B cells (88, 100, 119, 120, 239). Efficacy, however, is short-lived. Furthermore, systemic depletion and unwanted effects on protective immunity seen with anti-CD3 mAb therapy for instance, limit dosing and repeated application for efforts to extend β cell protection. On the other hand, antigen-based immunotherapy, such as β cell protein/peptide or particle vaccination, must contend with what is likely to be a high degree of diversity in the β cell-specific T cell response among T1D individuals and T1D subsets (199–201). Therefore, the ability to tolerize a sufficient number of disease-driving self-reactive T cell clones residing in the islets will be challenging. The most effective approach may reside in combining strategies functioning *via* distinct mechanisms. ND Ab specific for CD4 and CD8, for example, can be used to purge pathogenic Teff residing in the islets (128, 133). This would then be followed by an antigen-based strategy in which the need to establish a sufficiently sized pool of aTreg becomes less stringent. A novel combinatorial approach recently described utilizes IL-2C and MHC class II-peptide tetramers. This combination expands the frequency of Foxp3^+^Treg in a peptide-specific manner that results in prevention of diabetes in NOD mice (240).

Further studies defining common biomarkers of T1D will be critical in designing treatment protocols for individual patients, as well as for monitoring the effects of a given immunotherapy. Current advancements in large-scale genomic and proteomic analyses are making identification of these types of markers a reality, in addition to “matching” at risk or T1D patients with the most appropriate immunotherapy.

## Author Contributions

CJK, MC, QK, and RMT contributed to the preparation of the review article.

## Conflict of Interest Statement

The authors declare that the research was conducted in the absence of any commercial or financial relationships that could be construed as a potential conflict of interest.
